# How have contemporary studies evaluating self-help digital mental health interventions for young people monitored and reported on adverse events and harms?: a systematic scoping review

**DOI:** 10.3389/fpsyg.2026.1679968

**Published:** 2026-09-18

**Authors:** Sara Munir, Sophie Dallison, Harriet Padley, Hiu Man Fung, Grace Perry, Charlotte L. Hall, Maria E. Loades

**Affiliations:** 1 Department of Psychology, University of Bath, Bath, United Kingdom; 2 Academic Unit of Mental Health & Clinical Neurosciences, School of Medicine, University of Nottingham, Nottingham, United Kingdom; 3 NIHR MindTech MedTech Health Research Centre, Institute of Mental Health, University of Nottingham, Nottingham, United Kingdom; 4 NIHR Nottingham Biomedical Research Centre, Institute of Mental Health, University of Nottingham School of Medicine, Nottingham, United Kingdom

**Keywords:** digital mental health intervention, harms, mental health intervention safety, risk reporting, safety monitoring, young people

## Abstract

**Background:**

Many digital mental health interventions (DMHIs) can be effective in reducing symptoms of common mental health problems in young people and can overcome many barriers to accessing treatment. These are most scalable when offered as self-help. However, all mental health interventions have the potential to cause harm, and this should be monitored.

**Aim:**

To identify current practices and gaps in safety reporting, we systematically mapped how adverse events have been monitored and reported in contemporary studies evaluating self-guided DMHIs for young people.

**Method:**

We conducted a systematic scoping review. Two databases were searched for relevant studies published between 2019 and 2024. We synthesised the findings narratively, drawing on content analysis to identify patterns.

**Results:**

We included 37 studies, 12 (32.4%) of which described how they assessed adverse events (AEs). AEs were identified through worsening on wellbeing/psychometric measures, measures of risk and suicidal ideation/behaviour, participants self-identifying AEs, and parental reporting of AEs. Additionally, some studies reported on the proportion of participants experiencing AEs, with 8 (21.6%) studies reporting adverse events as an outcome. The most commonly occurring AEs reported were hospitalisation, treatment non-response, depressive symptoms, and significant worsening of symptoms (each 5.4% respectively). Other AEs reported were self-harm, substance misuse, anxiety symptoms, suicidal thoughts, suicide attempt and headaches (each 2.7% respectively).

**Conclusion:**

Despite the potential for adverse events to occur while young people are using self-guided DMHIs, our review highlighted a gap in the monitoring of and reporting of adverse events in these kinds of intervention studies.

## Highlights

While self-guided DMHIs for young people offer significant benefits and can overcome critical barriers to accessing treatment, there is evidence of inconsistent reporting of both the assessment of and occurrence of adverse events (AEs) in contemporary intervention studies.

## Introduction

Recent years have seen a surge in digital interventions targeting mental health and wellbeing (Lecomte et al., 2020), which are often more scalable than traditional, clinic based interventions and therefore could narrow the needs-access gap. Existing evidence indicates that digital mental health interventions (DMHIs) for common mental health problems that young people experience like anxiety and depression have better outcomes than control conditions (Lehtimaki et al., 2021). DMHIs are most scalable when provided in self-guided format, albeit that effectiveness varies considerably, and usage patterns show a tendency for disengagement and discontinuation (Lehtimaki et al., 2021). Due to the absence of interaction with a clinician or human supporter, it is unclear how best to monitor and report safety, including adverse events, occurring during self-guided DMHI use (Allan et al., 2024). Therefore, unique considerations for safety monitoring are indicated. To establish what current practice is, we aimed to identify if and how studies evaluating self-help DMHIs for common mental health problems in young people published in recent years have assessed and reported on adverse events.

Any psychological interventions can have unintended unfavourable consequences and adverse events which arise during the course of intervention studies, and these should be monitored and reported (Wolpert et al., 2015). Adverse Events (AEs) are ‘untoward medical occurrences’ and may include unintended or unwanted consequences and deterioration in existing symptoms or newly emergent symptoms (Purgato and Cortese, 2023). Serious Adverse Events (SAEs) are those that result in a significant risk to the individual or others and can involve death, disability or hospitalisation (Klein et al., 2024). The term ‘event’ is used as opposed to ‘effect’, where there is uncertainty as to whether the adversity has been caused by the intervention itself. The principle of non-maleficence states that any intervention can only be considered to be beneficial if it does not cause harm (Purgato and Cortese, 2023), meaning that all clinical researchers are ethically responsible for addressing potential harms and should monitor for and report adverse events (Papaioannou et al., 2021; Duggan et al., 2014; Klein et al., 2024; Klatte et al., 2023). AEs are relatively common, estimated to occur in more than 1 in 10 participants in randomised controlled trials (RCTs; Klatte et al., 2023). Therefore, papers in which researchers report on the outcomes of intervention studies should also include a description of how they monitored for adverse events and report on such outcomes.

AE monitoring and reporting in psychological interventions (including but not limited to DMHIs) tends to operationalised in the form of assessing change on indicators like symptoms (Linden, 2013) and monitoring for early termination of treatment (Parker et al., 2013). Despite a CONSORT extension specifically on reporting harms in RCTs (Junqueira et al., 2023), prior systematic reviews found that AEs are often not reported on, and definitions of AEs are not standardised (Papaioannou et al., 2021; Duggan et al., 2014), including in studies of young people (Lodewyk et al., 2023; Bottema-Beutel et al., 2021).

DMHIs are typically delivered through smart phone apps, web pages, wearable devices, virtual reality, video games or chat bots, and range from entirely self-guided to those integrated with clinician support (Mohr et al., 2013; Bevan Jones et al., 2023; Hollis et al., 2025). Self-guided interventions are inherently scalable and can be accessed privately and anonymously (Babbage et al., 2022) which overcomes barriers to help-seeking that young people face like long wait times and stigma (Aguirre Velasco et al., 2020; Radez et al., 2020). However, while guided interventions benefit from real-time human oversight, self-guided DMHIs rely solely on user input and automated processes. This absence of interpersonal interaction significantly limits the capacity to detect adverse events (AEs), particularly those that are not explicitly measured or voluntarily disclosed. Users may struggle to recognise or report negative experiences, and without targeted prompts or monitoring mechanisms, serious issues, such as symptom deterioration, suicidal ideation, or disengagement, may go unnoticed. Consequently, the detection and reporting of AEs in self-help DMHIs is both critically important and inherently difficult, underscoring the need for robust safety protocols and transparent reporting practices in this growing field.

Findings from prior systematic reviews reporting AE practice in DMHI studies have highlighted a particular gap in knowledge about monitoring practices for self-guided DMHIs in young people. A review which explored the reporting of AEs of DMHIs for any age group and any presenting emotional or behavioural disorder using trial registry database information to identify registered RCTs found that DMHIs delivered with human support were more likely to include a statement on AEs than those with only remote or no support, with only 50% of the latter including such a statement (Gomez Bergin et al., 2023). However, only a small number of the RCTs they included were aimed at young people. A young person focused review of RCTs found that while DMHIs show moderate efficacy for anxiety in children and adolescents, only one third of studies reported adverse effect outcomes, and of these, only 2 were self-guided DMHIs (Dulsen and Baumeister, 2024).

Given the specific challenges related to monitoring AEs in self-guided DMHI studies, and the specific vulnerabilities faced by young people, a review that focuses specifically on how AEs are assessed and reported in any DMHI evaluation reporting on outcomes in this population is sorely needed. Therefore, we aimed to depict how AEs, risks and harms have been investigated and reported on in contemporary intervention studies of self-help DMHIs aimed at treating depression and/or anxiety or enhancing the wellbeing of young people.

## Method

### Protocol and registration

The protocol for this review was pre-registered on the Open Science Framework^1^ before searches commenced.

### Design

We conducted a mapping review following a pre-defined framework to identify and synthesise relevant evidence (Campbell et al., 2020). Searches were carried out to identify published contemporary intervention studies reporting on outcomes of self-help DMHIs for young people. The focus of the current review is to answer questions relating to adverse events and harms monitoring and reporting. Other questions of interest will be reported elsewhere.

### Search strategy

We conducted a systematic search and study selection process to identify relevant papers meeting our prespecified inclusion criteria. Given the exploratory nature of a scoping review, our aim was to map the breadth of existing literature rather than exhaustively capture every study. We therefore selected two well-established, high-yield databases, *PubMed* and *APA Psycnet,* both of which are widely used in mental health and digital intervention research, ensuring a balance between comprehensiveness and feasibility. We also conducted supplementary searches using Google Scholar and the Cochrane database. We acknowledge that searching additional databases, including those covering related disciplines such as education, social sciences, or technology research, may have identified further relevant studies.

We borrowed from search terms devised by Babbage et al. in their review capturing DMHIs aimed at common mental health problems in young people, provided as self-help (Babbage et al., 2022). Our search concepts were: ‘adolescents’, ‘digital self-help’ and ‘common mental health problems/wellbeing’. MeSH terms, key terms and Boolean operators were used, in addition to search filters for publication date and human subjects research (see Supplementary material S1 for search terms).

### Eligibility criteria

We used an abbreviated form of the PICOS framework (Methley et al., 2014) to define our eligibility criteria, including PIS (population, intervention, study type), but not the ‘outcome’ and ‘comparator’ elements as they were not relevant.

The population was young people, therefore studies where participants were adolescents/young people aged 10–24 were included. However, where the mean age was >22 or <12, studies were excluded, as were studies where participants were predominantly under the age of 10 or over the age of 24. Studies where the participants also had physical health problems were included, providing that the main aim of the intervention being tested was to improve their mental health and wellbeing.

The intervention was any self-help DMHI (digital intervention aimed at addressing anxiety/depression symptoms, emotional problems or improving wellbeing, including interventions targeting related constructs like sleep and stress, as long as anxiety, depression or wellbeing were measured pre-post intervention). Digital format could include web-based, short message service (SMS)-based, or app-based, including interventions supported by artificial intelligence. We defined self-help as unguided interventions that are completed independently, and those interventions that had limited contact with a clinician/human were only included where the interaction was not part of the primary intervention, for example for onboarding, research or enrolment only.

The study type was any peer reviewed intervention study published since 2019. We focused on contemporary studies because the rapidly evolving functionality and formats of DMHIs not only influence the types of adverse events and potential harms that may arise, but also shape the ways in which these events can and should be monitored. We included all pre-post interventions studies which reported on quantitative outcomes, including RCTs, pilot RCTs, pre-post designs and case studies. Where studies were not published in English, were not peer-reviewed, and were published prior to 2019, these were excluded.

### Selection of studies

Following database searches (08/2024), studies were imported to Endnote for deduplication and then to Covidence for selection and extraction. Four members of the study team were involved in the screening process, all of whom were psychology students, including 2 doctoral researchers (SM, SD) and 2 undergraduate psychology students. Title and abstract screening were carried out independently for each study by two reviewers (SM and SD) to exclude irrelevant studies. Those articles that progressed to full text screening were then screened by either SM or SD, followed by 50% of papers being double screened by one of the two undergraduate students, so that each paper was screened twice. Thus, at both stages, each article was screened by at least one of the two doctoral students. At each stage, any conflicts were resolved by reviewers through discussion (which was also used as an opportunity to clarify the eligibility criteria and provide further training to less experienced members of the team) with input from a senior member of the study team (ML) where necessary to reach consensus. Reasons for excluding articles at full text screening stage were documented. Inter-rater reliability cannot be reported because a team of multiple reviewers with varied pairings prevented consistent tracking of conflicts.

### Data extraction

For all articles that met the inclusion criteria, data were extracted in Covidence. The data extraction form was piloted independently by two members of the study team (SM, SD). Following this, data were extracted from each study by one researcher and cross-checked against the article by a second researcher. Information we extracted included basic descriptive details about the study, clinical population, intervention, and adverse event assessment (method) and reporting (outcomes/findings). We used a list of types of AEs (see Supplementary material S2) which we created based on a prior systematic review that identified 27 types of adverse events and harms in intervention studies of young people (Lodewyk et al., 2023). Additionally, any data reflecting the narrative used to report the AEs (how AEs were reported and measured) was extracted.

### Data analysis

Covidence was used to generate summary tables. Studies were grouped by (1) whether they had assessed adverse events (yes/no), (2) reported adverse events (yes/no), (3) AE type based on the AE checklist and (4) the number of studies reporting each AE, respectively. Percentage and proportions were calculated. Subsequently, the narrative used to describe AE reporting/assessment was synthesised to illustrate how AEs were documented. As per the protocol, the text was coded to develop themes to synthesise similarities between studies (Thomas and Harden, 2008), however we deviated from our initial pre-specified plan as the data were not sufficiently rich to undertake a full thematic analysis. Instead, we drew upon content analysis to split the data into meaningful units (Mikkonen and Kääriäinen, 2020). *A priori* coding of the data took place to create categories, followed by two researchers independently organising the data into sub-categories, and listing the number of citations represented by each category (Stemler, 2000).

### Quality appraisal

In our pre-registered protocol, we specified the use of a critical appraisal tool. However, when we piloted a range of tools, including the Mixed Methods Appraisal Tool (MMAT), the categories assessed did not seem relevant to the questions or purpose of the current review. Given that we aimed to synthesise assessment and reporting of an outcome, rather than report on effectiveness, we concluded that formally assessing quality or risk of bias was not indicated (Munn et al., 2018), and we therefore did not use a quality appraisal tool. Instead, we specifically critique studies on adverse events assessment and reporting, which is our outcome of interest.

## Results

### Selection of studies and study characteristics

Figure 1 depicts numbers of papers progressing through each stage of the search and selection process. After full text screening, 37 papers, reporting on 37 separate studies met the inclusion criteria. Of these 19 studies (51%) were RCTs, 11 (30%) were feasibility/acceptability studies, 3 (8%) were randomised control feasibility studies, 2 (5%) used a quasi-experimental design, along with 1 (3%) open trial, and 1 (3%) non-randomised control study. An overview of studies is provided in Table 1.

**Figure 1:**
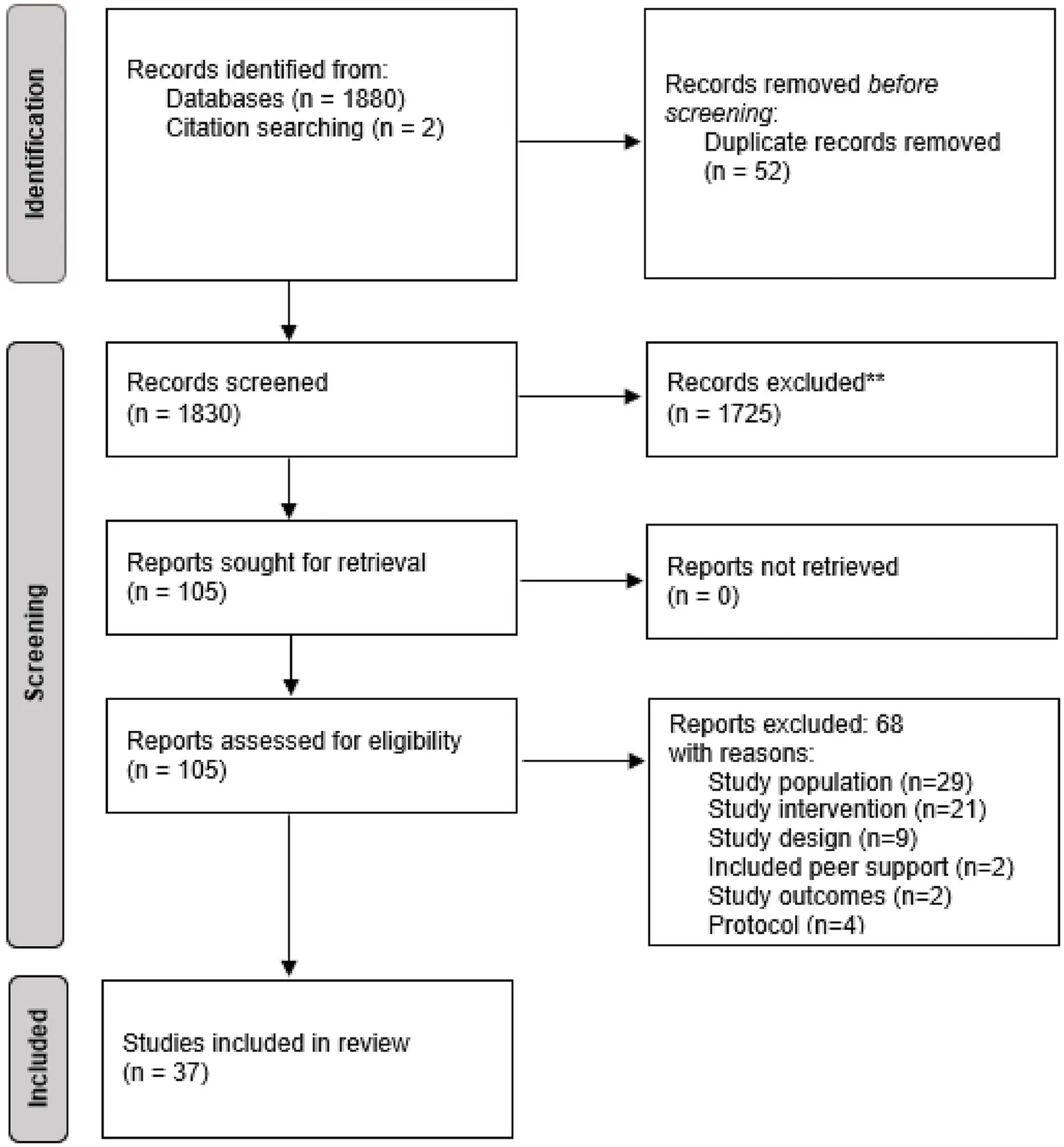
PRISMA flow diagram documenting the study selection process (Tricco et al., 2018).

**Table 1:** Description of included studies.

Authors and year	Country	Study design	Sample size; age range (mean); education level	Self-guided digital mental health intervention evaluated	AE assessment described in methods	AE findings reported in results
Abramovitch et al. (2024)	United States	Randomised Controlled Trial	70; 18–65 (18.85); university	OCD^a^.app - anxiety, mood and sleep: An app-based 2-week perfectionism intervention designed facilitate adaptive interpretations of thoughts, emotions and events associated with anxiety, mood and sleep problems, consisting of three gamified ‘levels’ a day.	No	No
Bevan Jones et al. (2020)	Wales	Feasibility/Acceptability Study (including pre-post measures)	36; 13–23 (16.3); school	MoodHwb: A multiplatform intervention designed to support young people with depression through psychoeducation, and self-management activities, with a section for parents and carers. Included illustrations, animations and interactive components.	No	No
Carmona et al. (2021)	Canada	Feasibility/Acceptability Study (including pre-post measures)	51; 15–24 (20.17); university, other	DOZE (Delivering Online Zzz’s with Empirical Support): A web-based app designed to address sleep disturbance among adolescents and young adults. Included a psychoeducation, a sleep diary, personalised feedback, and goal setting.	No	No
Chang et al. (2022)	United States	Randomised Controlled Trial	679; ^m^–^m^ (21.3); university	A 25-min web-based module teaching two Upa Yoga practices designed to improve student wellbeing during COVID-19. Participants were then provided with instructional videos and audio guidance and recommended to practice daily for 12 weeks.	No	No
Cook et al. (2019)	England	Randomised Controlled Trial	235; 18–24 (20); university	i-RFCBT (Internet-based Rumination-focused Cogntive Behavioural Therapy): A web-based 6-module CBT^b^ intervention to address rumination by shifting thinking to become more concrete, specific and action-orientated. Included psychoeducation, mood diaries, audio exercises, and testimonials. There were two versions: guided and unguided.	No	Yes
Crosby et al. (2023)	United States	Feasibility/Acceptability Study (including pre-post measures)	38; 18–24 (19.24); university	Sleep Scholar: A single-session, 3-module, self-guided, web-based insomnia intervention. Included sleep psychoeducation, stimulus control therapy, and sleep quality enhancement	Yes	No
Dobias et al. (2022)	United States	Randomised Controlled Trial	Project ABC = 3,679, Project SAVE = 1,652, The REFRAME = 848; 13–19 (Project SAVE = 15.71, other conditions = ^m^); unknown	Project ABC (Action Brings Change)^:^ A 5- to 8- min web-based single-session intervention to boost mood and build self-efficacy by encouraging engagement in pleasurable, value-aligned behaviours. Included psychoeducation, value assessment, planning and writing exercises. Project SAVE (Stop Adolescent Violence Everywhere): An 8-min web-based single-session intervention designed to reduce self-harm behaviours. Included psychoeducation, statistics and testimonials and alternative coping strategies. The REFRAME: A 5-min web-based single-session intervention teaching cognitive reappraisal. Included psychoeducation, testimonials, a practice exercise and prompts to apply cognitive reappraisal to their own lives.	No	No
Dodd et al. (2022)	United States	Randomised Controlled Trial	167; ^m^–^m^ (19); university	CBM-I (Cognitive Bias Modification Interpretation Retraining) task: A 2-session intervention for perfectionism involving brief scenarios with non-perfectionistic endings. Participants attended to the non-perfectionistic endings then visualised themselves in the scenario and imagined what would happen next.	Yes	No
Finlay-Jones et al. (2023)	Australia	Randomised Control Trial	151; 16–25 (21.15); unknown	A 4-week, self-guided, web-based self-compassion training program for improving the well-being of young people living with a chronic medical condition. Included psychoeducation, reflection and meditation exercises.	No	No
Fiodorova and Farb (2022)	Canada	Randomised Control Trial	105; ^m^–^m^ (18); university	A web-based 3-week intervention aiming to improve well-being. Involved a daily 5-min self-care reflection exercise using a mood board, stress rating and check-in questions.	No	No
Fitzpatrick et al. (2023)	United States	Randomised Control Trial	357; 11–14 (12.01); school	Project SOLVE: A 30-min, web-based, self-guided single-session intervention intended to reduce internalising symptoms by strengthening adolescents’ everyday problem solving. Included psychoeducation, vignettes and practice exercises.	No	No
Garbett et al. (2023)	Indonesia	Randomised Control Trial	1847; 15–19 (16.94); unknown	Warna-Warni Waktu: A single-session, social media-based intervention designed to reduce body dissatisfaction among young women, based on change techniques such as psychoeducation, media literacy and cognitive dissonance. Included videos telling a story of a woman navigating appearance pressures, with short interactive activities.	Yes	No
Harith et al. (2024)	Malaysia	Quasi Experimental	464; 18–25 (20.88); university	EduMind: A 7-day web-based intervention tailored to participants’ individual mental health status including psychoeducation, CBT^b^ elements, breathing exercises and mood journaling. Participants were encouraged to interact with the website for 10 min daily, at least five times a week.	No	No
He et al. (2022)	United States	Randomised Controlled Trial	148; 17–21 (18.78); university	XiaoE: A 7-day web-based artificial intelligence chatbot-guided CBT^b^ intervention for young adults with depression. The chatbot provided daily modules on topics such as cognitive distortions, self-esteem, mindfulness, loneliness and gratitude journaling.	No	No
Houck et al. (2022)	United States	Feasibility/Acceptability Study (including pre-post measures)	42; 12–14 (^m^); school	iTRAC (Internet-based Talking about Risk and Adolescent Choices): A 4-module, web-based intervention targeting sexual risk behaviours through emotional regulation skills training. Used a gaming task to increase emotional awareness and recognition and teach strategies for emotion regulation.	No	No
Jaycox et al. (2019)	United States	Feasibility/Acceptability Study (including pre-post measures)	51; ^m^–^m^ (15.02); school	LIFT (Life Improvement For Teens): A 7-module web-based CBT^b^ intervention for either stress or trauma (depending on the adolescents self-reported trauma symptoms at the start of the programme). Included interactive elements, gamified tasks, goal setting and mental health assessments.	No	No
Kahlon et al. (2023)	Norway	Randomised controlled trial	100; 13–16 (14.2); school	VRET (Virtual Reality Exposure Therapy): A self-guided 3-week virtual reality intervention for public speaking anxiety in adolescents. Involved speech exercises with virtual audience and gamified elements.	Yes	No
Kulikov et al. (2023)	United States	Randomised Control Feasibility Trial	51; 13–21 (intervention = 17.9; control = 16.96); school, university	Spark v2.0: A 5-week app-based BA^c^ intervention for depression in adolescents. Included psychoeducation on BA^c^, tracking features, skill building, identifying barriers and planning features.	Yes	Yes
Maciejewski and Smoktunowicz (2023)	Poland	Randomised Control Trial	372; 18–47 (20.98); unknown	Stressbot: A self-guided 7-day web-based intervention aiming to reinforce coping self-efficacy, reduce stress and improve quality of life. Involved 6 daily exercises delivered as messages which users could reply to via text, voice or buttons.	No	No
March et al. (2021)	Australia	Open trial	10,366; 7–17 (12.47); school	BRAVE Self-Help: A 10-session web-based self-guided CBT^b^ program for anxiety in young people. Included psychoeducation and activities such as relaxation training, cognitive restructuring, and graded exposure.	No	No
Miller et al. (2023)	United States	Feasibility/Acceptability Study (including pre-post measures)	30; 12–21 (17.03); unknown	Spark v1.0: A 5-week, self-guided BA^c^-based intervention for depression in adolescents. Psychoeducation and tasks BA^c^, mood tracking, mindfulness, activity scheduling problem-solving and relapse prevention were delivered through a mobile app, with corresponding virtual reality experiences through a headset.	Yes	Yes
Na et al. (2022)	Korea	Feasibility/Acceptability Study (including pre-post measures)	35; 18–25 (21.66); university	MEndorphins: A 7-day app-based BA^c^ intervention for stress self-management. Included monitoring current mood, planning stress management strategies, self-evaluation and sharing.	No	No
Osborn et al. (2020)	Kenya	Randomised Controlled Trial	103; 13–18 (−^m^); school	Shamiri-Digital: a web-based self-guided single-session intervention for depression, anxiety and mental wellbeing, consisting of three modules: growth mindset, gratitude, and value affirmation. Each module included psychoeducation and a related task.	No	No
Papini et al. (2023)	United States	Randomised Controlled Trial	159; ^m^–^m^ (19.27); university	AIM (Anxiety Insight Modules): A two-week CBT^b^-based anxiety psychoeducation intervention including interactive components and comprehension assessments.	No	No
Peake et al. (2024)	United States	Randomised Controlled Trial	160; 13–21 (intervention = 16.89, control = 16.79); unknown	Spark v2.1 and 2.2: A 5-week, self-guided BA^c^-based intervention for depression in adolescents. Provided psychoeducational content and delivered BA^c^ by teaching mood activity logging and activity scheduling.	Yes	Yes
Perumbil Pathrose et al. (2022)	Australia	Feasibility/Acceptability Study (including pre-post measures)	31; 14–29 (21.65); school, university	Mindfulness-based e-book: A web-based 6-module mindfulness intervention focusing on paying attention to the five senses and self-care. The modules incorporated audios, reflection activities, and interactive quizzes.	Yes	No
Radomski et al. (2020)	Canada	Randomised Controlled Trial	536; 13–19 (16.6); unknown	The Breathe program: A six-session web-based CBT^b^ intervention for anxiety, which included content such as relaxation, avoidance, cognitive distortions and realistic thinking. Included risk management, psychoeducation, reflection and activities.	Yes	Yes
Schleider et al. (2020)	United States	Feasibility/Acceptability Study (including pre-post measures)	539; 11–17 (−^m^); unknown	Project Personality: A self-guided, 30-min single-session intervention teaching how and why to adopt a growth mindset. Project CARE: A self-guided, 30-min single-session intervention teaching how and why to increase self-compassion to reduce self-hate. The ABC (Action Brings Change) Project: A self-guided, 30-min single-session BA^c^ intervention for depression teaching how and why we should engage in valued activities.	Yes	No
Serlachius et al. (2021)	New Zealand	Feasibility/Acceptability Study (including pre-post measures)	41; 16–30 (21.25); school, university	Whitu: An app-based 2-week CBT^b^- and Positive Psychology-based intervention for young people’s emotional well-being. Content included relaxation, self-compassion, gratitude and goal setting.	No	Yes
Smith et al. (2023)	Unites States	Feasibility/Acceptability Study (including pre-post measures)	74; 13–17 (−^m^); unknown	Project Body Neutrality; A web-based, self-guided single-session intervention that aims to increase body functionality appreciation to reduce body dissatisfaction in young people. Included psychoeducation, self-reflection exercises and writing prompts.	No	No
Subotic-Kerry et al. (2023)	Australia	Quasi Experimental	336; 12–15 (13.08); school	The Bite Back Mental Fitness Challenge: A 7-module, web-based, self-guided Positive Psychology intervention aiming to encourage self-reflection and personal growth. Included animations, questionnaires and activities.	No	Yes
Suffoletto et al. (2021)	United States	Randomised Controlled Trial	52; 18-^m^ (18.7); university	MoST-MH (Mobile Support Tool for Mental Health): A 3-month text message- and web-based intervention aimed to improve mental health using Positive Psychology, CBT^b^ and DBT^d^ strategies. Involved increasing awareness of current symptoms, stressors and their impact, and boosting self-efficacy.	No	No
Theurel et al. (2022)	France	Non-randomised Controlled Trial	103; 18–25 (intervention = 20.9, control = 19.8); university	ETUCARE; an 8-week, web-based intervention designed to improve university students’ mental health during COVID-19 using strategies from CBT^b^, Positive Psychology, mindfulness and lifestyle medicine. Included psychoeducation and activities on topics such as stress management, procrastination, sleep and emotion regulation.	No	No
Torok et al. (2022)	Australia	Randomised Controlled Trial	455; 18–25 (21.5); unknown	Lifebuoy: a 7-module, app-based self-guided DBT^d^ intervention designed to improve emotional regulation and increase distress tolerance skills. Included psychoeducation, interactive exercises and mindfulness, breathing and self-soothing tools.	Yes	No
Watkins et al. (2024)	United Kingdom, Germany, Spain and Belgium	Randomised Controlled Trial	2,532; 16–22 (19.2); school, university	An app-based intervention for mental wellbeing. There were two versions: one based on CBT^b^ principles and strategies, and one focused on emotional competence. They both included psychoeducation, self-monitoring, strategies, practise exercises, and gamified aspects.	No	Yes
Wright et al. (2019)	United Kingdom	Randomised Controlled Trial	139; 12–18 (intervention = 14.9, control = 15.1); unknown	Stressbusters: An 8-session computer-based CBT^b^ program for depression. Each session is an interactive presentation featuring videos, animations, graphics, printouts and homework tasks.	Yes	No
Zelihić et al. (2022)	Norway	Randomised Controlled Trial	71; 11–18 (13.98); school	YPF (Young Persons Face): A 7-session self-guided web-based intervention designed to increase psychosocial adjustment to having an appearance-affecting condition. Included advice, guidance and strategies for managing common challenges related to having a visible difference.	No	No

^a^OCD, obsessive compulsive disorder; ^b^CBT, cognitive behavioural therapy; ^c^Behavioural Activation; ^d^Dialetic Behavioural Therapy; ^m^ mean; − not stated.

### Adverse event assessment

Of the 37 included studies, 12 (32.4%) described adverse events assessments (where the assessment of AEs is specified), see Table 1 for details. Various methods of assessing AEs were described by studies, including by deterioration on mental health or wellbeing measures, asking questions about suicidal ideation/behaviour, and allowing participants to self-identify adverse events, each of which we describe below with illustrative examples.

### Wellbeing/mental health symptom measures

In total, 10/37 (27%) studies utilised wellbeing/psychometric measures to measure AEs. For example, “Participants completed the short version (15 items) of the MFQ20 at the beginning of each session to monitor mood and assess risk” (Wright et al., 2019); “the research staff consulted with study clinicians if patient symptoms declined to a clinically significant degree (an increase in PHQ-8 score…)” (Miller et al., 2023).

### Questions about risk, suicidal ideation/behaviour

Five out of 37 studies (13.5%) used measures of risk and suicidal ideation to determine the presence of AEs; “If participants exceeded cutoff scores of 21 or greater on the Suicidal Ideation Attributes Scale (SIDAS), the research team was alerted” (Torok et al., 2022); “Check-in involved adolescents…indicating whether they had thoughts of self-harm or harming others” (Radomski et al., 2020).

### Participants self-identify AEs to research team

Two of 37 studies (5.4%) reported the assessment of AEs through participants self-reporting distress (Osborn et al., 2020; Serlachius et al., 2021), with the onus being on the participant to let study team know if they were distressed rather than there being a routinely used question or measure given to all to monitor the presence of AEs. For example, “Students were informed that they could talk to the study staff should they be distressed” (Osborn et al., 2020).

### Parental involvement in AE monitoring

Two of 37 studies (5.4%) involved parents in reporting AEs experienced by young people participants (Kulikov et al., 2023; Peake et al., 2024); “All participants and their legal guardians (for those aged >18 years) were asked to complete a weekly internally developed symptom check questionnaire that asked them to report any negative symptoms or side effects” (Peake et al., 2024).

### Adverse event reporting

Of the 37 included studies, 8 (21.6%) reported on adverse events outcomes (AEs that occurred during study participation), see Table 1 for details. Of these 8 studies, 6 reported the number of participants who had experienced AEs. For example, “For the 5% (4/80) of adolescents who reported that their anxiety was worse after the program, the average worsening rating was 1.3 (mostly same/hardly worse)” (Radomski et al., 2020); “There were a total of 2 AEs in the control arm and 4 in the Spark arm.” (Peake et al., 2024). Some studies gave more specific details in the results section, for example, “Three participants reported feeling overwhelmed by some of the reflective content of the app, namely being intimidated by the ‘Goal Setting’ module, feeling worse by not being able to name 3 support people in the ‘Connect’ module, and feeling unpleasant about having to reflect on negative memories in the ‘Be Kind to Yourself’ module.” (Serlachius et al., 2021).

The adverse events that were identified and most reported as AEs that arose during the study were hospitalisation, treatment non-response, increased depressive symptoms, and significant worsening of symptoms (each reported by 2 studies). Other AEs, each of which was reported as arising in 1 study, were self-harm, substance misuse, anxiety symptoms, suicidal thoughts, suicide attempt and headache. Table 2 depicts the number of studies reporting AEs in each AE category.

**Table 2:** Adverse events that were reported in included studies categorised using an adverse events list developed by Lodewyk et al. (Lodewyk et al., 2023).

AE category	No. studies (%)	Citation
Hospitalisation	2 (5.4%)	Kulikov et al. (2023); Watkins et al. (2024)
Self-harm	1 (2.7%)	Watkins et al. (2024)
Substance misuse	1 (2.7%)	Watkins et al. (2024)
Anxiety symptoms	1 (2.7%)	Radomski et al. (2020)
Treatment non-response	2 (5.4%)	Radomski et al. (2020); Cook et al. (2019)
Depressive symptoms	2(5.4%)	Kulikov et al. (2023); Peake et al. (2024)
Suicidal thoughts	1 (2.7%)	Peake et al. (2024)
Significant worsening of symptoms	2 (5.4%)	Kulikov et al. (2023); Peake et al. (2024)
Headache	1 (2.7%)	Miller et al. (2023)
Suicide attempt	1 (2.7%)	Kulikov et al. (2023)

### Reporting no AEs without evidence of AE assessment

The outcome reporting of 3/37 studies (8%) suggests that no AEs occurred during the studies, however no detail is provided in the methods section of those studies about how AEs were assessed. For example, “there have been no reported adverse effects among those using mindfulness interventions in nonclinical and clinical populations” (Perumbil Pathrose et al., 2022). “There was no clear evidence of potential negative effects from using the program” (Bevan Jones et al., 2020). Although AE assessments may have taken place, this was not clearly reported. See Table 3 for further details.

**Table 3:** Content analysis of adverse event monitoring and reporting.

Category	Sub-category	Frequency of studies in each category	Citations
1. Measuring of adverse events	Use of wellbeing/psychometric measures for AE monitoring	(10)	Wright et al. (2019); Miller et al. (2023); Radomski et al. (2020); Kulikov et al. (2023); Peake et al. (2024); Dodd et al. (2022); Kahlon et al. (2023); Schleider et al. (2020); Subotic-Kerry et al. (2023); Watkins et al. (2024)
	Measures of risk, suicidal ideation/behaviour for AE monitoring	(5)	Wright et al. (2019); Torok et al. (2022); Radomski et al. (2020); Peake et al. (2024); Cook et al. (2019)
	Participants self-identify AEs by direct contact or focus groups only (let study team know if in distress rather than formal measure)	(2)	Osborn et al. (2020); Serlachius et al. (2021)
	Parental involvement in AE monitoring		Kulikov et al. (2023); Peake et al. (2024)
2. Reporting of adverse events	Proportion of ppts with AEs listed	(6)	Radomski et al. (2020); Serlachius et al. (2021); Kulikov et al. (2023); Peake et al. (2024); Subotic-Kerry et al. (2023); Watkins et al. (2024)
	Reporting no AEs without evidence of AE assessment	(3)	Perumbil Pathrose et al. (2022); Bevan Jones et al. (2020); Chang et al. (2022)

AEs, Adverse events; Ppts, participants.

## Discussion

Our scoping review found that only a minority (32.4%) of studies evaluating digital self-help interventions for young people described how they assessed adverse events (AEs), and even fewer (21.6%) reported AEs as outcomes. Reported AEs included hospitalisation, treatment non-response, depressive symptoms, and significant symptom worsening.

We identified substantial variability and inconsistency in how AEs are monitored and reported in self-help DMHI studies. This heterogeneity reflects, in part, the considerable diversity of interventions included in this review, which ranged across different therapeutic approaches, technologies, target populations, and levels of support. The nature and likelihood of potential harms may therefore differ substantially between interventions, meaning that not all AEs can be expected to be captured by a single standardised approach. Furthermore, some potential AEs are specific to digitally delivered interventions generally (such as increased screen time) or to specific types of digital interventions (such as virtual reality delivered interventions which could result in cyber-sickness). Nevertheless, a core set of harms should be consistently monitored across interventions, alongside intervention-specific harms where relevant, given that even minor harms, when scaled across populations, could have significant consequences (Foulkes et al., 2023).

Beyond the diversity of interventions, what constitutes the most appropriate AE assessment needs to be considered in relation to both the study design and the population of interest. For instance, we found that symptom deterioration was used as an AE indicator in some studies, but the interpretation of symptoms worsening will depend on study design. In uncontrolled studies, deterioration cannot necessarily be attributed to the intervention and may instead reflect the natural course or fluctuation of symptoms over time (Hankin et al., 2025). In controlled studies, comparisons between intervention and control groups provide a stronger basis for assessing whether deterioration is plausibly intervention-related. In relation to the population of interest, developmental stage is important as differences in cognitive, emotional, and social development across children, adolescents, and young adults may influence both the experience and reporting of adverse events, highlighting the need for age-appropriate approaches to harm monitoring.

The lack of assessment or reporting of AEs found in our review aligns with previous findings in paediatric mental health trials, where AE monitoring and reporting is similarly underreported (Lodewyk et al., 2023). However, the self-guided nature of the interventions we focused our review on presents unique challenges for AE monitoring. Without human interaction, researchers cannot observe participants directly or respond to unreported distress. The sole reliance on self-disclosure assumes emotional literacy and willingness to report, which may not always be present. Thus, relying on self-report may be insufficient to capture the full range of potential harms (Rozental et al., 2014). Involving parents or caregiver, especially for younger adolescents, could enhance AE detection, yet this was rarely done.

Beyond the challenge of how best to monitor AEs in the context of self-guided DMHIs, several other factors may contribute to underreporting. Researchers may assume that self-guided interventions are inherently low risk because they are brief, accessible and do not involve direct therapeutic contact. They may also fear that AE monitoring could undermine participant engagement or increase distress (Wolpert, 2016). Practical constraints, such as limited follow-up, participant anonymity, and journal word limit, could also play a role (Junqueira et al., 2023; Phillips et al., 2019). Expectations from journals and funders regarding the reporting of harms may also be less well established than expectations for reporting intervention benefits. Despite these challenges, the anonymity and accessibility of DMHIs make them attractive to young people who may otherwise avoid support due to stigma (Radez et al., 2020), reinforcing the need for robust safety monitoring.

Furthermore, a lack of consensus on AE definitions and classifications additionally complicates reporting (Allan et al., 2024; Klein et al., 2024) and not having standardised guidance on what constitutes an AE in digital mental health research, and how AEs should be monitored and reported, is likely to be an important contributing factor. Existing typologies vary widely, from direct and psychological harms to equity and opportunity costs (Linden, 2013; Papaioannou et al., 2024; Lorenc and Oliver, 2014). This inconsistency likely contributes to the diverse and often limited AE monitoring methods observed in our review. Linked to this is the absence of guidance on how to monitor AEs. In our review, 10 studies used wellbeing or psychometric measures to assess AEs, five included risk or suicidality measures, and four relied on self- or parent-reported AEs. While tools like the PANEPS-I scale (Baumeister et al., 2024) show promise, they may not capture the full spectrum of harms relevant to DMHIs and further work is needed to co-design appropriate measures in partnership with young people that are fit for purpose in self-guided DMHIs.

Regardless of the cause, the lack of measuring and reporting of AEs in main outcomes papers is problematic, because it prevents readers from making an informed assessment of the benefits and potential risks of an intervention. More critically, it may mean that an intervention contains unrecognised harms, and is unknowingly unsafe (Rozental et al., 2015).

### Strengths and limitations

Conclusions drawn from this review must be interpreted alongside several limitations. Our databases searches were conducted in August 2024. Most included studies have taken place in middle-high income countries, which limits the generalisability of our findings. As we relied on published papers, the included studies may not fully represent the complete evidence base, given that studies with null findings may be less likely to be published. By relying on published reports, our findings reflect reported rather than actual adverse event monitoring practices, and it is possible that some studies undertook monitoring procedures that were not fully described in their publications. Furthermore, limiting our primary search to only two databases (with additional supplementary searches on Google Scholar and the Cochrane database) may have resulted in the exclusion of relevant studies, potentially narrowing the scope of our findings. However, given that our aim was to map current practices rather than to identify the most comprehensive or highest-quality evidence, we considered this approach appropriate for the objectives of a scoping review. Therefore, the reported percentages are descriptive of the included studies and should not be interpreted as estimates of adverse event monitoring or reporting practices across the wider literature.

We did not complete a general quality assessment or risk of bias assessment and our included studies are likely variable in methodological rigour (Munn et al., 2018). However, this is not particularly relevant to answering our key questions of interest in this review, which pertains specifically to AE monitoring and reporting. Questions about sample size or recruitment strategy, for instance, were therefore not deemed useful but we did specifically critique studies in detail on their methodology for monitoring AEs and their reporting of these as our outcomes of interest.

By including studies from 2019 to 2024, we have provided a clear picture of the current state of AE monitoring and reporting in self-help DMHI research in young people. We have included all DMHIs that aimed to enhance wellbeing and mental health; therefore, our pool of studies reflects a wide range of interventions. This means that our findings are generally applicable but there may be particular considerations pertinent to AE monitoring and reporting for specific conditions or interventions. For example, a self-help DMHI which makes use of virtual reality would need to monitor and report on side effects and potential harms specific to that technology like cybersickness and eye strain. Future research should further examine and synthesise qualitative evidence to better understand participants’ perspectives on potential harms associated with digital mental health interventions. As most of the included studies have taken place in middle-high income countries, the generalisability of our findings is limited and more work in low-income countries is much needed.

We have been transparent in our search strategy and data extraction process, to ensure that our research is replicable. Searches were conducted by two reviewers as well as study selection, data extraction and validation being completed independently by two reviewers. This increased reliability and reduced the risk of selective reporting.

### Clinical and research implications

The systematic assessment of AEs in young people’s mental health interventions would require little change to data collection (for example, monitoring deterioration psychometric measures to monitor AEs or introducing an additional AE measurement to pre-existing measures administered during the trial), but could mean a major change in the way the data are utilised and formulated (Wolpert et al., 2015), and would enhance young people’s safety. Furthermore, given the range of potential harms associated with digital interventions, monitoring approaches should extend beyond symptom deterioration to capture both psychological outcomes and broader harms specific to the intervention, technology, and context of delivery. Based on our findings, we recommend considering two parts of Wolpert et al.’s three-level framework for considering AEs and harms in self- guided DMHIs for young people (Wolpert et al., 2015). This framework was designed to assess harm from youth mental health intervention and suggests the evaluation of: (1) *Harm caused by ineffective engagement*: young people who drop out before completing the recommended amount of treatment; (2) *Harm caused by ineffective practise*: measuring the implementation of a model of care, and (3) *Harm caused by adverse events*: measuring of unfavourable outcomes of interventions. During self-guided DMHIs however, we would not expect for the model of care to be deviated from, therefore the second factor is not necessary in this context. We also recommend considering Lodewyk et al.’s work, which suggests a checklist of AEs based on prior research, as well as open-ended questions to identify unanticipated AEs (Lodewyk et al., 2023).

Future research should develop and test standardised methods for assessing and reporting adverse events (AEs) in young people, potentially by integrating Wolpert’s framework with Lodewyk et al.’s AE checklist (Wolpert et al., 2015; Lodewyk et al., 2023). Guidance should clarify definitions of AEs and harms, distinguish expected symptom changes from potential intervention-related deterioration, and set minimum standards for monitoring and reporting. Harms should be reported alongside benefits in main outcomes papers, rather than only when serious events occur. We recommend that both young people and parents are encouraged to report concerns, using a structured, standardised approach. Policymakers should mandate such practices, especially given the growing reliance on self-guided DMHIs to address gaps in mental healthcare provision. AE checklists should also be expanded to capture subtler effects, such as changes in social behaviour or academic performance. Real-time monitoring through apps and wearable devices, tools young people are generally open to using, could support this effort (Knight and Bidargaddi, 2018).

This review provides an evidence base for informing the development of such guidance by demonstrating both the extent of current reporting gaps and the diversity of approaches currently used. Developing consensus-based recommendations with researchers, young people and families, clinicians, ethics committees, trial oversight bodies, funders, journals and policymakers could help establish proportionate expectations for safety monitoring across different types of digital mental health intervention. Such guidance should recognise that digital interventions are not uniformly low risk, while also avoiding disproportionate monitoring requirements that could unnecessarily limit access to potentially beneficial low-intensity support.

## Conclusion

Our review highlights a critical gap in the monitoring and reporting of adverse events in contemporary self-help DMHI research for young people. Given the growing reliance on these interventions, researchers must clearly describe AE assessment methods and report all outcomes, both of effectiveness and potential for harm, to support informed decision-making and safeguard user wellbeing.

## Footnotes

1
https://doi.org/10.17605/OSF.IO/2RFWX
