# Supplementary material for: How have contemporary studies evaluating self-help digital mental health interventions for young people monitored and reported on adverse events and harms?: a systematic scoping review
Source: Front Psychol. 2026 Sep 18;17:1679968. doi: 10.3389/fpsyg.2026.1679968 (PMC13631862; doi:10.3389/fpsyg.2026.1679968)
Supplement: Supplementary file 1 [file Supplementary_file_1.DOCX]

**Online Supplementary Materials**

*S1.Search terms for each database*

**Search terms for PubMed**

| **Column  Terms**  **Combined with** | **Adolescents**  **AND** | **Common mental health/wellbeing**  **AND** | **Digital**  **AND** | **Self-help** |
| --- | --- | --- | --- | --- |
| **OR** | Adolescent [Title/Abstract] OR  Adolescence [Title/Abstract] OR  Adolescents [Title/Abstract] | Anxiety [Title/Abstract] | E-health [Title/Abstract]  Ehealth [Title/Abstract]  E health [Title/Abstract] | Self help [Title/Abstract]  Self-help [Title/Abstract]  Selfhelp [Title/Abstract] |
| **OR** | Teen [Title/Abstract] OR  Teenager [Title/Abstract] OR  Teens [Title/Abstract] | Anxiety [mh] | Telemedicine [mh] | Self-help groups [mh] |
| **OR** | Youth [Title/Abstract]  Youths [Title/Abstract]  Youthful [Title/Abstract] | Anxiety Disorders [mh] | Computer Intervention [Title/Abstract]  Computer interventions [Title/Abstract] | Self-management [mh] |
| **OR** | Minor [Title/Abstract]  Minors [Title/Abstract] | Anxiety Disorder [Title/Abstract]  Anxiety disorders [Title/Abstract] | Computer assisted instruction [mh] | Self-care [mh] |
| **OR** | Child [Title/Abstract]  Children [Title/Abstract] | Stress [Title/Abstract] | Therapy, Computer-Assisted [mh] | Self-management [Title/Abstract]  Self management [Title/Abstract]  Selfmanagement [Title/Abstract] |
| **OR** | Young person [Title/Abstract]  Young people [Title/Abstract]  Young persons [Title/Abstract] | Stress Disorders [mh] | Health promotion [mh] | Self-care [Title/Abstract]  Self care [Title/Abstract]  Selfcare [Title/Abstract] |
| **OR** | Adolescent [mh] | Stress, psychological [mh] | Internet intervention [Title/Abstract]  Internet interventions [Title/Abstract] | Guided [Title/Abstract] |
| **OR** | Child [mh] | Stress, Physiological [mh] | Internet | Guided self-help  [Title/Abstract] |
| **OR** | College student  [Title/Abstract]  OR  College student  [Title/Abstract] | Affective Disorders [Title/Abstract] | Digital health intervention [Title/Abstract]  Digital health interventions [Title/Abstract] | Modules  [Title/Abstract] |
| **OR** | University student  [Title/Abstract]  OR  University students  [Title/Abstract] | Mood Disorders [mh] | Online intervention [Title/Abstract]  Online interventions [Title/Abstract] | Self help techniques  [Title/Abstract]  OR  Self-help techniques  [Title/Abstract] |
| **OR** | Young adult  [Title/Abstract]  OR  Young adults  [Title/Abstract] | Mental Disorders [mh] | Mobile application [Title/Abstract]  Mobile applications [Title/Abstract] |  |
| **OR** | Student  [Title/Abstract]  OR  Students  [Title/Abstract] | Mood [Title/Abstract] | Medical Informatics Applications [mh] |  |
| **OR** | Student (mh) | Affect [mh] | Tablet intervention [Title/Abstract]  Tablet interventions [Title/Abstract] |  |
| **OR** |  | Affective symptoms [mh] | Electronic Health [Title/Abstract]  Electronic-health [Title/Abstract] |  |
| **OR** | Young adult (mh) | Depression [Title/Abstract] | Digital intervention [Title/Abstract]  Digital interventions [Title/Abstract] |  |
| **OR** |  | Depression [mh] | Computer Systems [mh] |  |
| **OR** |  | Wellbeing [Title/Abstract]  Well-being [Title/Abstract]  Well being [Title/Abstract] | Telephone [mh] |  |
| **OR** |  | Distress [Title/Abstract] | Wireless Technology [mh] |  |
| **OR** |  | Mental health  [Title/Abstract] | Electronic intervention [Title/Abstract]  Electronic interventions [Title/Abstract] |  |
|  |  | Mental health {mh) | Online  [Title/Abstract] |  |
| **OR** |  | Psychopathology  [Title/Abstract] | Digital  [Title/Abstract] |  |
| **OR** |  | Psychopathology (mh) | Computerised  [Title/Abstract] |  |
| **OR** |  | Mental wellness  [Title/Abstract] | Technology-based intervention  [Title/Abstract]  OR  Technology based intervention  [Title/Abstract] |  |
| **OR** |  | Mental illness  [Title/Abstract] | Telehealth  [Title/Abstract] |  |
| **OR** |  | Mental disorder  [Title/Abstract] | mHealth  [Title/Abstract] |  |
| **OR** |  | Internalising  [Title/Abstract]  OR  Internalizing  [Title/Abstract]  OR  Internalising disorder  OR  Internalizing disorder | eHealth   [Title/Abstract] |  |
| **OR** |  | Psychiatric disorder  [Title/Abstract] | Web-based  [Title/Abstract]  OR  Web based  [Title/Abstract] |  |
| **OR** |  | Psychiatric illness  [Title/Abstract] | Internet-based  [Title/Abstract]  Internet based  [Title/Abstract] |  |
| **OR** |  | Reduced wellbeing [Title/Abstract] | Mobile phone  [Title/Abstract] |  |
| **OR** |  | Disorder  [Title/Abstract]  OR  Disorders  [Title/Abstract] | Text message  [Title/Abstract]  OR  Text messaging  [Title/Abstract] |  |
| **OR** |  | Worry  [Title/Abstract] | Text messaging (mh) |  |
| **OR** |  |  | SMS  [Title/Abstract] |  |
| **OR** |  |  | Cell phone  [Title/Abstract]  OR  Cellular phone  [Title/Abstract] |  |
| **OR** |  |  | Cell phone (mh) |  |
|  |  | **AND** | **AND** | **AND** |

***Search terms for APA PsychInfo***

| **Column Terms**  **Combined with** | **Adolescents**  **AND** | **Common mental health problems/wellbeing AND** | **Digital**  **AND** | **Self help** |
| --- | --- | --- | --- | --- |
| **OR** | Adolescen* | Anxiety | E?health | Self?help |
| **OR** | Teen* | Anxiety (MeSH) | Telemedicine (MeSH) | Self-help groups (MeSH) |
| **OR** | Youth* | Anxiety Disorders (MeSH) | Computer Intervention* | Self-management (MESH) |
| **OR** | Minor* | Anxiety Disorder* | Computer assisted instruction (MESH) | Self-care (MeSH) |
| **OR** | Child* | Stress | Therapy, Computer-Assisted (MESH) | Self?management |
| **OR** | Young pe* | Stress Disorders (MESH) | Health promotion (MeSH) | Self?care |
| **OR** | Adolescent (MeSH) | Stress, psychological (MeSH) | Internet intervention* | Guided |
| **OR** | Child (MeSH) | Stress, Physiological (MESH) | Internet | Guided self?help |
| **OR** |  | Affective Disorders | Digital health intervention* | modules |
| **OR** | College student | Mood Disorders (MeSH) |  | Self-help techniques |
| **OR** | University student | Mental Disorders (MESH) | Online intervention* |  |
| **OR** | Young adult | Mood |  |  |
| **OR** | Student | Affect (MESH) | Mobile application* |  |
| **OR** |  | Affective symptoms (MESH) | Medical Informatics Applications (MeSH) |  |
| **OR** |  | Depression | Tablet intervention* |  |
| **OR** |  | Depression (MESH) | Electronic?Health |  |
| **OR** |  | Well?being | Digital intervention* |  |
| **OR** |  | Distress | Computer Systems (MeSH) |  |
| **OR** |  | Mental health | Telephone (MeSH) |  |
| **OR** |  | Psychopathology | Wireless Technology (MeSH) |  |
| **OR** |  | Mental wellness | Electronic intervention* |  |
| **OR** |  | Mental illness | Online |  |
| **OR** |  | Mental disorder | Digital |  |
| **OR** |  | Internalising | Computerised |  |
| **OR** |  | Internalizing | E?therapy |  |
| **OR** |  | Reduced wellbeing | Technology based intervention |  |
| **OR** |  | Worry | Telehealth |  |
| **OR** |  |  | mHealth |  |
| **OR** |  |  | eHealth |  |
| **OR** |  |  | Web?based |  |
| **OR** |  |  | Internet?based |  |
| **OR** |  |  | Mobile phone |  |
| **OR** |  |  | Text message |  |
| **OR** |  |  | SMS |  |
| **OR** |  |  | Cell phone |  |
|  |  | **AND** | **AND** | **AND** |

*S2. – Adverse Events Checklist (Lodewyk et al., 2023)*

1. sleep disturbances
2. restlessness/overactivity
3. stomach ache
4. weight gain
5. nausea
6. breathing problems
7. headache
8. sweating
9. anxiety symptoms
10. depressive symptoms
11. suicide attempt
12. mood problems
13. anger/tantrums
14. obsessions/compulsions
15. significant worsening of symptoms
16. hospitalisation
17. treatment non-response
18. missed time in school
19. social withdrawal
20. life-threatening events
21. event that results in significant disability
22. self-harm resulting in several physical injury
23. important medical events
24. severe exacerbation of symptoms
25. suicidal thoughts

Reference:

Lodewyk, K., Bagnell, A., Courtney, D. B., & Newton, A. S. (2023). Adverse event monitoring and reporting in studies of pediatric psychosocial interventions: a systematic review. *Child Adolesc Ment Health*, *28*(3), 425-437.
